# Identifying Gaps in Mobile Data Collection by Frontline Health Workers in Bangladesh

**DOI:** 10.5334/aogh.4868

**Published:** 2025-10-13

**Authors:** Monzur Morshed Patwary, Naimul Islam

**Affiliations:** 1 Emory University, Atlanta, GA 30322, United States; 2 FHI 360, Durham, NC, United States

**Keywords:** mHealth, community health workers (CHWs), community health, data, mobile health

## Abstract

*Background:* Mobile health (mHealth) tools are replacing paper-based surveys for frontline health workers, promising speed and cost-effectiveness. Yet, in low- and middle-income settings, there is scope for research on the accuracy of the information captured.

*Objectives:* To assess the quality of socio-demographic and economic data collected through the mHealth platform by BRAC (the largest non-profit in Bangladesh) Shasthya Kormis or SKs (frontline health workers), and to identify reasons for data gaps.

*Methods:* A mixed-methods study (2021) analyzed secondary mHealth records for 388 households drawn via two-stage cluster sampling from the catchment areas of 30 randomly selected SKs working across 61 districts. Descriptive statistics in R quantified missing values and irregular entries in household registration, visits, and member forms. Complementary insights were obtained from 24 in-depth interviews with SKs; transcripts were thematically coded using an iteratively refined codebook.

*Findings:* Core demographic variables were largely complete, but considerable gaps persisted: national ID/birth ID (84% missing), phone numbers (77%), household assets (39–70%), and land-size data. Several explanations were deduced: reluctance of community members to share sensitive information, sometimes to secure social benefits; recall or estimation difficulties for ages and land measurements; and operational barriers: poor connectivity, offline notetaking, and syncing errors that deterred submission in a timely manner.

*Conclusions:* While mHealth simplifies nationwide community data collection in this case, data quality is affected by social hesitancy, recall bias, and technical issues.

## Introduction

Mobile health (mHealth) technologies have steadily become indispensable to data collection by frontline health workers worldwide as they considerably decrease the costs and inconveniences associated with paper-based surveys. Mobile-based data collection brings myriads of benefits, such as improvement in timeliness and completeness of data, decreased error rates, and greater cost-effectiveness compared to traditional methods [1]. mHealth interventions have contributed to enhanced technical performance and higher satisfaction among health workers, suggesting promise for strengthening health systems worldwide [2].

Digital health initiatives in Bangladesh have generally received strong support at the highest political level [3]. The practice of mHealth data collection from the communities in the country began through the “Mobile Lady” project by DNet [4]. The largest NGO (non-governmental organization)-led implementation of mHealth in the country took place through the initiative of BRAC, a Bangladesh-based global NGO. Its health program deployed an mHealth app in 2020 to digitize its nationwide data collection by its extensive network of frontline health workers (Shasthya Kormis, SKs). The organization’s mHealth platform allows 4,300+ SKs in Bangladesh to register households, track longitudinal health data, and provide targeted services. The platform supports integration between community and facility data, real-time performance monitoring, and rapid updates, which collectively improve efficiency and decision-making [5]. Armed with an android device containing mHealth app, the SKs conduct door-to-door visits to digitally collect household and member data. During these visits, they complete multiple forms, such as, eligible couple, pregnancy care, and NCD care. Based on the registered member database, they provide age-appropriate counseling and services (i.e., NCD management, vision care, etc.) to target populations.

Sound decisions are based on sound data; therefore, it is essential to ensure that the data are of good quality.

The Global Digital Health Strategy 2020–2025 from the World Health Organization underlines the importance of data quality as informed decisions are based on sound data [6]. Dependable and up-to-date health information is crucial for the success of public health efforts to maintain routine service provision, respond to outbreaks, and implement prevention measures. Evidence-based approaches to mHealth implementation highlight the sheer importance of data quality and user-centered design in ensuring effective results [7]. Data accuracy, especially in this era, remains a pivotal aspect of public health information quality, as errors in electronic health data have the capacity to distort aggregated data used for public health decision-making [8]. Furthermore, data quality issues like incompleteness and inaccuracy can decrease trust in public health data and can negatively affect health program performance [9].

This study discusses issues faced by SKs most frequently when they collect sociodemographic and economic data leveraging the mHealth app.

## Methods

The study, conducted in 2021, looked at household data from 61 districts in Bangladesh (where BRAC operated at that time), collected and recorded in mHealth by BRAC SKs. It entails a multi-method approach by analyzing secondary mHealth data to determine frequently occurring issues and also taking interviews with SKs to get qualitative insights about those issues. Informed consent was obtained before each interview. Quantitative analyses were conducted with the R programming language, and Microsoft Excel was used for the qualitative data display matrix.

Those under the inclusion criteria were: SKs and members of the household from communities who interacted with SKs in the past two months during their household visits.

Aiming to achieve a balance between accuracy, efficiency, and cost-effectiveness, we leveraged cluster sampling to select the households. Initially, we randomly picked 30 SKs out of 4,162, and subsequently 30 households in each of their catchment areas were picked using systematic sampling. Furthermore, three households were selected for each Shasthya Shebikas (volunteers reporting to SKs). The total sample size came down to 900, where 388 households received follow-up household visits. For the quantitative analysis, the final sample size was 388 households. For qualitative research, we aimed to conduct in-depth Interviews in Bengali (the local language) with SKs at the field level. We reached data saturation after conducting 24 such interviews. 

The analysis entailed quantitative assessment of mHealth secondary data quality and errors and qualitative thematic analysis of interview data. For the qualitative part, we developed an initial codebook with a priori codes from first-phase findings, including definitions and application guidelines. Following transcription of the interviews, we conducted thematic analysis, and emergent codes were added to the codebook during analysis.

Data were organized using a display matrix containing quotations representing sub-codes within each major code. Related codes were then clustered into overarching themes for results presentation.

For this study, we focused exclusively on household visits, household registration, and member forms. Prior to data collection, we reviewed these forms and verified their logical connections with other forms. The forms follow a dependent structure where both member registration and household visit forms are linked to the household registration form. This creates a mandatory sequence needing SKs to complete household registration before getting access to the other two forms.

This study was approved by the BRAC James P Grant School of Public Health and the BRAC Health Program. Approval covered secondary analysis of de-identified mHealth records and in-depth interviews. Written and oral informed consent was obtained from all interview participants. For the secondary dataset, all records were de-identified prior to analysis.

## Results

Secondary data analysis revealed considerable missing values and data irregularities across a number of indicators. Missing values seemed to be especially high for household financial status (38%), NID or birth ID (84%), asset information (39–70%), and household contact phone numbers (77%). Additionally, irregular values were spotted in household member counts and cultivable/homestead land measurements.

To further explore these data quality issues, we interviewed 24 SKs, asking questions to gauge their knowledge and perception of how to complete the three forms.

**Figure 1:**
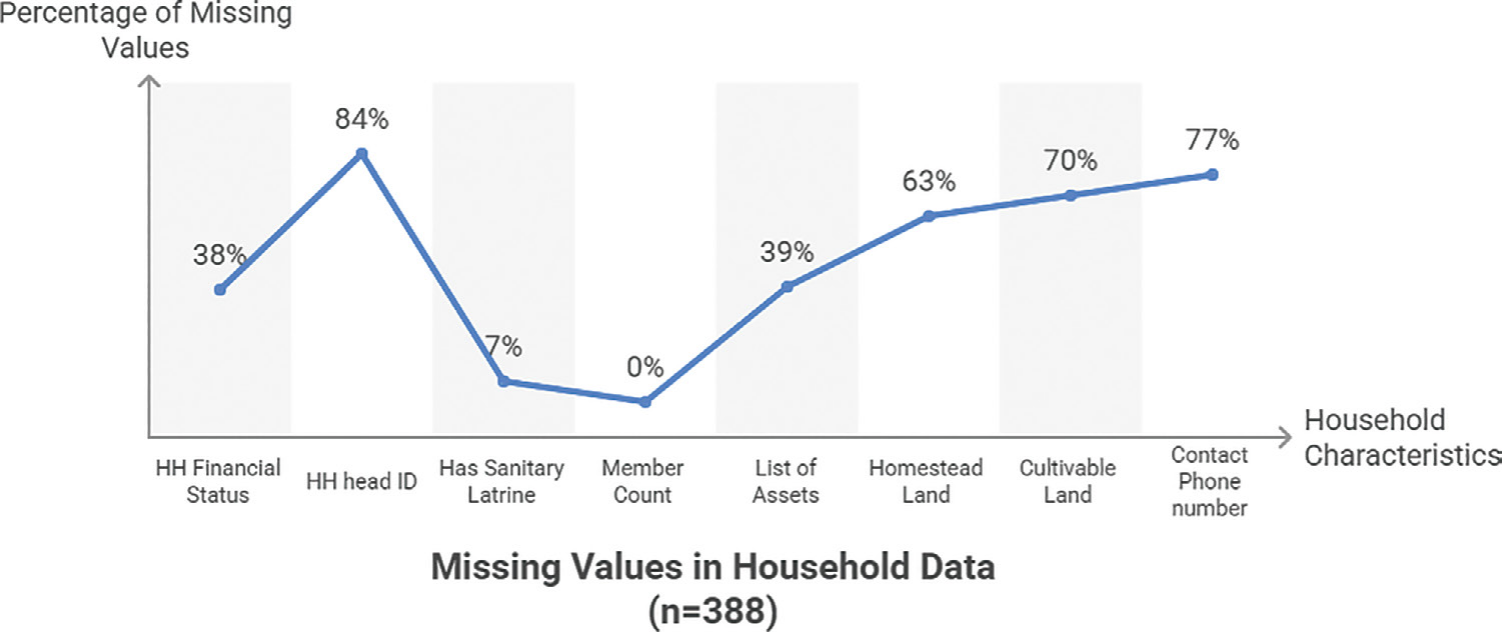
This graph shows the missing values (across different indicators) in the data collected by Shasthya Kormis.

**Figure 2:**
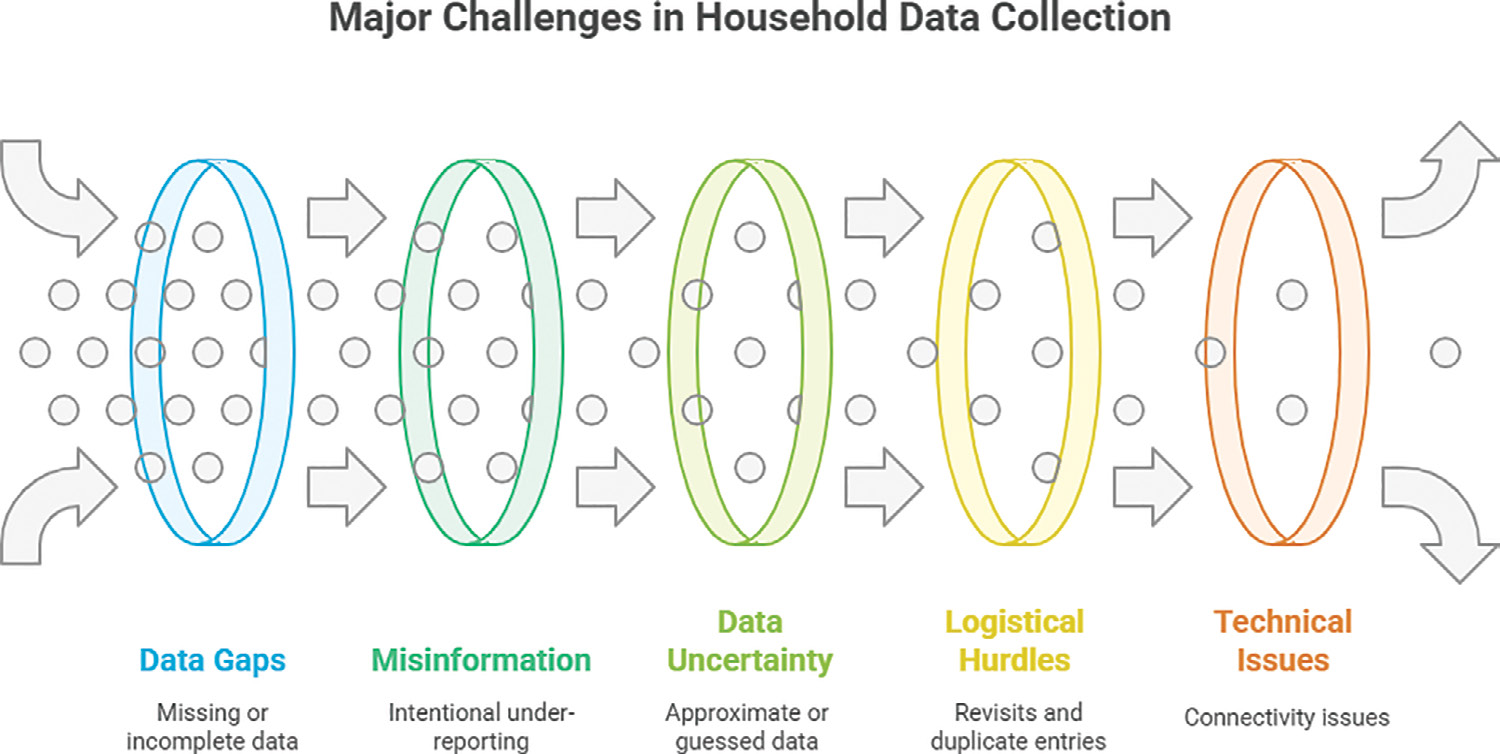
This graph illustrates the most prominent quality issues faced by Shasthya Kormis during data collection.

Findings indicated that SKs possessed adequate knowledge of data collection requirements for household visit forms (Figures 1 and 2). They reported regularly updating household information during follow-up visits, including sociodemographic, economic, and sanitation data, as well as member-specific details such as identification, personal information, household head relationships, and disability status. SKs routinely updated member lists to reflect births and deaths, and could add new households when discovering previously unregistered families or household divisions within their catchment areas. Interestingly, they did not report using the migration tracking features on the app despite having access.

Although SKs are aware that they need to get the NID (Bangladeshi national identity card) and mobile phone numbers, they often leave them out because someone is absent or does not agree to share. SK Nur Ayesha remarked, “The men are out in the fields, and the women could not provide their numbers, so we have to settle with incomplete data.” Mahmuda echoed her colleague, saying how sometimes it is not possible to get a hold of the correct mobile phone numbers in even two or three visits. Sraboni, like many of her peers, told us, “Some people under-report the amount of land they own to be eligible for benefits. This is essentially false information.” Morium Begum, pointing at guesstimates from the community members, recalled, “They can’t give an exact age, and we have to record accordingly.” According to SKs, issues in data entry were also attributed to system errors. For instance, Juma Das said, “Without internet connectivity, we are not able to submit; so we write it on paper and later type it up.” Her colleague, Shahanaj remarked, “The dashboard report does not add up, not everything we type in the app gets reflected on the web.”

## Conclusion

In Bangladesh, there exists a number of issues involving data collection by frontline health workers, with this study shedding light on the reluctance among community members to share personal information. NGOs can consider sensitizing the community on the value of socio-demographic and public health data, as well as re-evaluating data collection protocols to ensure complete, accurate data from the community.

It was not in the scope of this study to identify feasible interventions for improving data accuracy. This necessitates the need for substantial focus on data quality issues and evidence-based solutions. Enhanced training and motivation programs for frontline health workers such as SKs are essential to ensure robust data quality standards.

The study’s generalizability is limited considering the small sample size, minimizing wider applicability to community populations, health workers, and data systems. Nevertheless, our findings share important insights into the challenges inherent in collecting socio-demographic and economic health data at the community level, especially involving technological platforms.

The results may allow stakeholders like donors and implementers to plan informed large-scale socio-demographic health data collection initiatives in low- and middle-income country contexts.
